# Does trappability and self-selection influence cognitive performance?

**DOI:** 10.1098/rsos.220473

**Published:** 2022-09-14

**Authors:** Benjamin J. Ashton, Alex Thornton, Elizabeth M. Speechley, Amanda R. Ridley

**Affiliations:** ^1^ School of Natural Sciences, Macquarie University, Sydney, New South Wales 2109, Australia; ^2^ Centre for Evolutionary Biology, School of Biological Sciences, University of Western Australia, Perth, Western Australia 6009, Australia; ^3^ Centre for Ecology and Conservation, University of Exeter, Penryn Campus, Treliever Road, Penryn TR10 9FE, UK

**Keywords:** cognition, STRANGE, sampling bias, Australian magpie, associative learning

## Abstract

Recent research has highlighted how trappability and self-selection—the processes by which individuals with particular traits may be more likely to be caught or to participate in experiments—may be sources of bias in studies of animal behaviour and cognition. It is crucial to determine whether such biases exist, and if they do, what effect they have on results. In this study, we investigated if trappability (quantified through ‘ringing status’—whether or not a bird had been trapped for ringing) and self-selection are sources of bias in a series of associative learning experiments spanning 5 years in the Western Australian magpie (*Gymnorhina tibicen dorsalis*). We found no evidence of self-selection, with no biases in task participation associated with sex, age, group size or ringing status. In addition, we found that there was no effect of trappability on cognitive performance. These findings give us confidence in the results generated in the animal cognition literature and add to a growing body of literature seeking to determine potential sources of bias in studies of animal behaviour, and how they influence the generalizability and reproducibility of findings.

## 1. Introduction

It has been 10 years since Henrich *et al.* [1] highlighted that biased sampling from Western, Educated, Industrialized, Rich and Democratic (WEIRD) societies undermine the field of human psychology. It is increasingly clear that similar biases may affect research on animal behaviour and cognition [2–6]. Webster & Rutz [2] describe a framework where sampling biases may occur as a result of Social background, Trappability and self-selection, Rearing history, Acclimation and habitat, Natural changes in responsiveness, Genetic make-up, and Experience (STRANGE). Such biases have the potential to undermine the generalizability and reproducibility of results—accordingly, it is crucial to determine if such sampling biases occur, and if they do, what effect they have on the conclusions drawn from studies.

Trappability may be a source of bias if trapping selects for individuals with certain personality traits, such as boldness, sociability or dominance [7,8]. Indeed, there is evidence suggesting that variation in behaviour is related to capture probability—for instance bold individuals are more likely to be trapped than risk-averse individuals [7,8]. Similarly, self-selection, or the process by which individuals with particular traits are more likely to participate in experiments, may also be a source of bias in studies of animal behaviour. Evidence from the primate literature indicates that participation in experiments is biased toward specific personality traits, such as openness and assertiveness [6]. Accordingly, field studies of animal behaviour risk underrepresenting natural variation in trait phenotypes due to biases associated with trappability and self-selection. This is of particular concern if differences in trait phenotypes translate to differences in experimental performance.

In particular, the fields of cognitive ecology and cognitive evolution have the potential to be influenced by sampling biases. A number of recent studies using psychometric tasks to investigate the causes and consequences of individual variation in cognitive performance have generated important insights into cognitive evolution. Studies have found links between individual cognitive performance and aspects of the social [9,10] and physical environment [11,12], and measures of fitness [9,13,14]. Considerable effort has been made to ensure that measures of cognitive performance are robust, with studies attempting to control for potentially confounding factors such as stimulus salience, motivation, neophobia, social learning and order effects [15–17]. In addition, the repeatability of cognitive performance has been investigated in a number of species to determine whether measures of performance are reliable and not determined by extraneous non-cognitive factors that are likely to vary over time (such as energetic state and persistence). To this end, a recent meta-analysis found moderate support for temporal and contextual repeatability of cognitive performance [18]. Furthermore, short- and long-term repeatability of cognitive performance has been identified in Australian magpies [9,19]. However, the potential influence of sampling biases on cognitive performance has been largely overlooked.

In this study, we investigated whether trappability and self-selection influence the conclusions drawn from a long-term project investigating the causes and consequences of individual variation in associative learning performance in the Western Australian magpie (*Gymnorhina tibicen dorsalis*). A number of cognition studies have been carried out on this study population [9,15,20,21]; however, because studies have focused on ringed individuals (who are individually recognizable due to the unique ring combinations), there is a risk of biased sampling toward individuals with certain personality traits. Currently, it remains unknown if trappability and self-selection is a source of bias in this study population. This study aims to examine if such a bias exists, and if it does, whether it influences cognitive performance.

## 2. Methods

### 2.1. Study species and population

This study was conducted on a wild population of Western Australian magpies in the Guildford and Crawley suburbs of Perth, Western Australia. The Western Australian magpie is a large (250–400 g), sexually dichromatic, cooperatively breeding bird occurring in territorial groups of 3–12 individuals [22]. The study population is comprised of an average of 86 individuals (range 70–122) from 18 groups annually. The population has been continuously monitored since 2013 and has undergone several studies involving cognitive testing [9,15,20,21,23]. Although the majority of individuals in the study population are ringed, groups contain both ringed and unringed individuals. Unringed individuals can be identified by sex (if there is only one unringed individual of the same sex in a given group) and diagnostic features on bills and/or plumage. For example, distinctive patterns or markings on the black plumage of the breast, or markings on the bill, can be used to identify unringed individuals. We did not test unringed juveniles from one year to the next due to identification problems associated with changes in plumage.

### 2.2. Cognitive testing

One hundred and ten free-living magpies (*N* = 99 adults (individuals > 3 years old), and *N* = 11 juveniles; *N* = 74 ringed individuals, and *N* = 36 unringed individuals) were presented with associative learning tasks between 2015 and 2020 (some of these data have been published previously [9,19]). Attempts were made to test all individuals within a group, regardless of whether they were ringed or not, reducing experimenter bias in selection of test subjects. Associative learning was chosen because this is a well-studied, ecologically relevant cognitive trait thought to underpin a wide range of fitness-relevant behaviours, from foraging strategies and predator–prey interactions to mate choice [24]. General cognitive performance (of which associative learning performance is a component) is positively related to the average number of hatched clutches per year, the average number of fledglings per year, and the average number of fledglings surviving to independence per year in female Australian magpies [9]. In addition, previous research on this study population (using a component of this dataset) has shown that several proxies of motivation (including latency to interact with the task and time interacting with the task) do not influence cognitive performance.

Associative learning was quantified by measuring the number of trials it took test subjects to learn an association between a colour shade and a food reward. This was achieved by presenting individuals with a foraging grid containing two wells covered with two lids of different shades of the same colour (2015 = dark blue/light blue, 2016 = dark green/light green, 2018 = dark purple/light purple, 2020 = dark pink/light pink and dark grey/light grey). Shades of colours, rather than distinct colours, were used to reduce the chances of prior experience influencing cognitive performance [17]. Furthermore, by using the same testing paradigm, but presenting causally identical but visually distinct tasks, we ensured that the same cognitive trait was quantified in each block of testing, while controlling for potentially confounding effects of memory for specific colours. During each testing period, one of the colour shades was randomly chosen to be the rewarded well for the duration of the experiment. A food reward could be accessed by test subjects pecking at the rewarded lid and retrieving a small amount of mozzarella cheese (see Ashton *et al*. [9] for training procedures). Test subjects were allowed to search both wells in the first trial to demonstrate that only one well was rewarded, but in all subsequent trials the task apparatus was removed after the first well was chosen. This ensured there was a cost associated with an incorrect choice. We made sure that individuals had searched the well of choice (rewarded or otherwise) before moving to remove the apparatus to prevent ‘Clever Hans’ effects. There was at least one minute between trials and a maximum of 50 trials per individual per day. To avoid social learning and social interference, all trials were carried out when individuals were over 10 m away from other group members. This was achievable as magpies often naturally forage this far apart [9]. Trials were terminated if another individual encroached within this distance before a well had been pecked. Previous work using unrewarded ‘probe’ trials indicates that magpies' performance in spatial memory tasks is not influenced by olfactory cues [9]. In the current task, unrewarded trials could potentially interfere with learning of the stimulus–reward association, so we controlled for olfactory cues by wiping both wells with cheese before trials. To ensure that colour was the cue being associated with the food reward and not the side of the grid, it was ensured that the side of the rewarded well was not the same for more than three consecutive trials. Individuals were considered to have passed the task when they chose the rewarded well in 10 out of 12 consecutive trials (this represents a significant deviation from binomial probability; binomial test, *p* = 0.039). The number of trials taken to reach this criterion (including the correct trials) was the measure of cognitive performance.

### 2.3. Statistical analyses

Self-selection: to determine if task participation was predicted by specific factors we ran a series of generalized linear mixed models (GLMMs) with task participation (yes/no) fitted as the binomial response term. An individual was classified as having not participated if a testing attempt was made but the bird did not engage with the task at all. All individuals that participated in at least one trial completed the task (i.e. reached criterion). Ringing status (whether an individual was ringed or not), age (juvenile or adult), and group size were included as explanatory terms. Group identity, individual identity (nested within group identity), observer ID and year tested were included as random terms.

Trappability: to determine the effect of trappability on cognitive performance we ran a series of GLMMs with a Poisson distribution. The number of trials taken to pass the task was included as a response term. Ringing status was used as a proxy for trappability (because an individual must have been trapped in order to have been ringed), and was included as an explanatory term. Prior to ringing we carried out ‘trap habituation’, whereby we placed a baited trap in the vicinity of the test subject but made no attempt to trap it. Following this, a baited walk-in trap was placed in the vicinity of the focal subject a second time—if the bird entered the back of the trap a closing mechanism was released and the bird was caught, ringed and released. At least one trapping attempt was made on all test subjects—accordingly, ringing status was deemed a suitable proxy for trappability. Age and group size were also included as explanatory terms. Group identity, individual identity (nested within group identity), observer ID and year tested were included as random terms.

An additional two analyses were carried out on a subset of individuals of known sex to determine the effect of sex on self-selection (*N* = 105 individuals) and cognitive performance (*N* = 79 individuals) respectively. The same modelling approach as the previous analyses was used.

In all analyses a model selection approach was adopted—following Burnham & Anderson [25], we used a hypothesis-driven approach to test the variables in question and compared them to a null model. Models were ranked in order of their AICc values (the lowest AICc value having the greatest explanatory power [25]). If a model was more than two AICc units smaller than any other model, then this was judged to explain the observed relationship in the data better than any other model. If there was more than one model with ΔAICc < 2 from the ‘best’ model, and predictor terms in that model had confidence intervals that did not intersect zero and explained more variation than the basic model (the model containing no predictors, just the constant and the random terms), then model averaging was carried out on this top set of models [26]. All statistical analyses were performed in R (v. 4.1.0; http://www.r-project.org), with the glmer function in the lme4 package [27].

## 3. Results

### 3.1. Self-selection

Between 2015 and 2020 we attempted to test 110 test subjects (in one to five different testing blocks—see Methods). Thirty test subjects failed to participate in at least one testing block and 93 test subjects participated in at least one testing block (these values do not add up to 110 as some test subjects participated in some blocks but not others). A total of 285 testing attempts were made—240 of these were successfully completed while 45 were not (84.2% completion rate). All 45 incomplete testing attempts were due to lack of participation—if a trial was interrupted an additional trial was presented when it was appropriate to do so (i.e. when the interrupting individual had moved away). This additional trial was considered the same testing attempt. There was no evidence of self-selection operating—none of the predictors tested explained differences in task participation between individuals (table 1 and electronic supplementary material, table S1).

**Table 1.** Factors influencing task participation in an associative learning task in Western Australian magpies (*N* = 110 individuals from 18 groups, *N* = 285 tests). An intercept model plus three models with one fixed effect each (group size, age and ringing status) were ran. Individual identity, group identity, observer and year tested were included as random terms. Models were ranked in order of their AICc values (the lowest AICc value having the greatest explanatory power).

model	AICc	ΔAICc	effect size	95% confidence intervals
ringing status	187.9	0		
unringed			−1.581	−3.696, 5.344
ringed^a^			0^a^	
intercept only	188.3	0.4		
group size	189.6	1.7	−0.196	−5.922, 2.000
age	190.2	2.3		
juvenile			−0.614	−3.606, 2.379
adult^a^			0^a^	

^a^This category's effect size is held to zero to allow comparison with other categories.

### 3.2. Trappability

**Figure 1.**
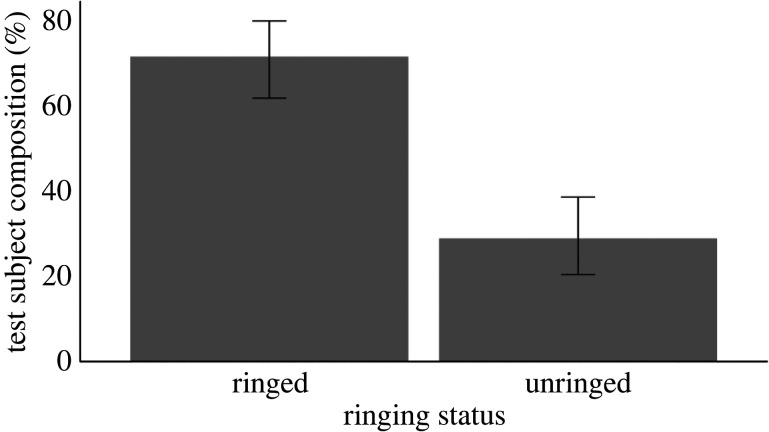
The composition of test subjects that completed cognitive testing, in terms of ringed versus unringed individuals (*N* = 67 ringed individuals, *N* = 27 unringed individuals). Error bars represent 95% confidence intervals. Although skewed towards ringed individuals, when taking into account the proportion of ringed versus unringed individuals in the whole study population (67.3% ringed individuals), it is apparent that self-selection is not operating.

The majority of individuals in the study population were ringed (*N* = 74 ringed individuals, 67.3% of study population; *N* = 36 unringed individuals, 32.7% of study population). However, a binomial test (accounting for study population bias, with the expected test proportion set at 0.673) found that the composition of individuals who completed cognitive testing was not significantly biased toward ringed individuals (binomial test, *p* = 0.240, figure 1, *N* = 67 ringed individuals, *N* = 27 unringed individuals), providing further evidence that self-selection was not operating with respect to trappability.

**Figure 2.**
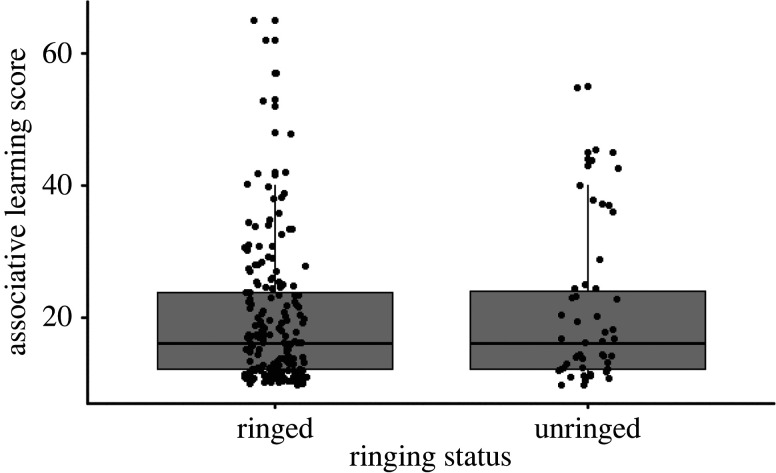
Performance on an associative learning task (number of trials taken to pass) in ringed and unringed Australian magpies. Higher score equals poorer performance.

Two hundred and forty associative learning tests were completed; 190 by ringed individuals and 50 by unringed individuals. We found no effect of ringing status on cognitive performance (table 2 and figure 2)—ringed (mean ± SE = 19.38 ± 0.761) and unringed (mean ± SE = 20.86 ± 1.674) individuals took a similar number of trials to pass the associative learning tasks. As per previous findings [9], the strongest predictor of cognitive performance was group size, whereby individuals from larger groups performed better than individuals from smaller groups (table 2). Age (table 2) and sex (electronic supplementary material, table S2) did not influence cognitive performance.

**Table 2.** The effect of ringing status on associative learning performance in Western Australian magpies (*N* = 84 individuals from 18 groups completing 240 tests). An intercept model plus three models with one fixed effect each (group size, age, and ringing status) were ran. Individual identity, group identity, observer and year tested were included as random effects. Models were ranked in order of their AICc values (the lowest AICc value having the greatest explanatory power).

model	AICc	ΔAICc	effect size	95% confidence intervals
group size	1736.2	0	−0.063	−0.093, −0.031
intercept only	1748.2	12		
age	1748.9	12.7		
juvenile			0.156	−0.103, 0.415
adult^a^			0^a^	
ringing status	1750.2	14		
unringed			−0.019	−0.194, 0.152
ringed^a^			0^a^	

^a^This category's effect size is held to zero to allow comparison with other categories.

## 4. Discussion

We found no significant predictors of task participation, indicating self-selection was not operating in the associative learning experiments carried out on the Australian magpies. The composition of the study population was biased toward ringed individuals, a result of methodological necessity to allow individual identification, but controlling for the difference in the number of ringed and unringed individuals, we found no evidence that ringed individuals were more likely to engage with cognitive tasks. Crucially, we found no effect of ringing status on cognitive performance, indicating trappability-related biases would not undermine the generalizability and reproducibility of findings derived from this dataset. However, it should be noted that although we tested 85% of the study population, it is possible that a characteristic common to the untested 15% of individuals precluded cognitive testing. It is therefore possible that an unidentified bias might influence task participation and experimental performance.

It may be hypothesized that unringed individuals are less likely to participate in experiments due to differences in personality. In order to ring birds it is necessary to trap them—accordingly, unringed individuals may be more ‘trap shy’ compared to ringed individuals. Therefore, unringed individuals may be more risk-averse compared to ringed individuals, and less likely to approach cognitive tasks. However, the current results suggest trappability does not influence task participation in Australian magpies (contrary to evidence from the primate literature [6]). While trappability might be a good indicator of certain personality traits (such as boldness and exploratory behaviour), it is necessary to compile multi-dimensional measures of such traits, from multiple measurements [6], and apply these to the self-selection framework, in order to comprehensively explore the relationship between personality and self-selection. Future work should also aim to make multiple trapping attempts in order to determine the repeatability, and obtain robust measures of, trappability. A limitation of this study is that trapping attempts were only made once on some individuals. Unlike previous studies, we found no evidence that sex [4,28] or age [28,29] influenced task participation. However, it is worth noting that we were only able to treat age as a categorical variable because the age of some birds was unknown (some individuals were already adults when we started our research on the study population)—inclusion of age as a continuous variable may have revealed different patterns of participation. Group size also did not affect task participation, indicating that group size-related participation biases are unlikely to confound previously identified relationships between group size and cognitive performance in the Australian magpie [9,16,21]. An increasing number of studies are using automated cognitive testing, for example through RFID systems that detect and record engagement and performance in cognitive tasks [30–32]. This approach to cognitive testing can generate large sample sizes and may eliminate certain biases—for example, the lack of an experimenter during testing may influence test subject engagement. Further investigation is required to determine how different experimental approaches may be more or less susceptible to biases in cognitive ecology.

Evidence that exploratory behaviour [33] and neophobia [34] influence cognitive performance may lead one to predict that differences in performance may arise due to trappability. However, previous work has found that neophobia does not differ between ringed and unringed Australian magpies, and there is no effect of neophobia on cognitive performance [9]. Despite this, it is noteworthy that ringed individuals did not perform differently compared to their unringed counterparts. That cognitive performance appears not to be influenced by trappability suggests that the task in question is providing robust measures of cognitive performance, and is not influenced by some of the confounding variables identified by the STRANGE framework.

Moving forward, it is important to determine how prevalent sampling biases are. If biases are identified in previously published studies, the potential effect of the bias(es) needs to be determined, and the interpretation of the data carefully reconsidered. Where possible, future studies should be designed such that the chances of biases operating are minimized. For example, this can be achieved by testing subjects that are representative of the species as a whole in terms of social background and genetic make-up, and testing across periods where experimental responsiveness may vary. Where it is not possible to eliminate biases, data that may help determine the potential effect of them should be collected—for example, testing history or trapping status can be incorporated into analyses to help determine the effect of experience or trappability on experimental performance. Where it is not possible to either control for or determine the effect of biases, the potential effect of the biases should be discussed explicitly.

## Data Availability

The data are provided in electronic supplementary material [35].
